# Associations of Trauma Severity with Mean Platelet Volume and Levels of Systemic Inflammatory Markers (IL1*β*, IL6, TNF*α*, and CRP)

**DOI:** 10.1155/2016/9894716

**Published:** 2016-04-05

**Authors:** Baris Alper, Baris Erdogan, Mehmet Özgür Erdogan, Korkut Bozan, Murat Can

**Affiliations:** ^1^Department of Emergency Medicine, Klinikum Fürth Zentrale Notaufnahme, 90766 Fürth, Germany; ^2^Department of Otolaryngology, Medipol University Healthcare and Research Center Esenler Hospital, 34230 Istanbul, Turkey; ^3^Department of Emergency Medicine, Haydarpasa Numune Training and Research Hospital, 34668 Istanbul, Turkey; ^4^Department of Emergency Medicine, Göztepe Medicalpark Hospital, 34732 Istanbul, Turkey; ^5^Department of Biochemistry, Bulent Ecevit University Hospital, 67600 Zonguldak, Turkey

## Abstract

We investigated the associations of injury severity scores (ISSs) with the mean platelet volume, the serum levels of two interleukins (IL1*β* and IL6), and the serum levels of tumour necrosis factor-*α* (TNF*α*) and C-reactive protein (CRP). We sought to identify biochemical parameters that could be used as components of a new biochemical parameter-based ISS system. The levels of CRP, TNF*α*, IL1*β*, and IL6 differed significantly (all *p* values < 0.05) between severely injured patients and controls. The mean platelet volume (MPV) did not correlate with the ISSs (*p* > 0.05). The TNF*α* and IL6 levels were useful for determining the severity of injury, and the CRP level was elevated in all trauma patients but did not correlate with the ISS. The IL1*β* level was higher in the study group but did not increase as the ISS increased. IL6 and TNF*α* levels were higher in the study group and increased as the ISS increased. We found no significant difference between the trauma group and healthy individuals in terms of MPV values. IL6 and TNF*α* levels can be used to assess trauma severity. However, neither the MPV nor the CRP or IL1*β* level is useful for this purpose.

## 1. Introduction

Trauma is the most common cause of death in young individuals, and it is the third most common cause of death for individuals of all ages [1]. Half of all deaths caused by trauma occur at the scenes of accidents; the rest occur within the following hours or days [2, 3]. Thus, early evaluation of such patients and correct determination of trauma severity are crucial.

Trauma-scoring systems have been developed to manage such patients effectively, but the current systems do not feature biochemical markers despite the fact that several blood markers are associated with trauma severity and mortality. In this prospective work, we sought to identify correlations between the injury severity score (ISS), on the one hand, and the mean platelet volume (MPV) and levels of C-reactive protein (CRP), interleukin (IL)1*β*, IL6, and tumour necrosis factor-alpha (TNF*α*), on the other. We tried to identify biochemical parameters that could serve as components of a new biochemical parameter-based ISS system.

## 2. Materials and Methods

### 2.1. Human Experimental Protocol

#### 2.1.1. Study Groups

This study was performed between 15 March 2014 and 15 July 2015 in the Haydarpasa Numune Training and Research Hospital, Emergency Department, after the Hospital Ethics Committee approved the research. All included patients provided written informed consent. A total of 84 patients with multiple traumas, aged 18–65 years, were included. Age, gender, blood pressure, heart rate, date and time of admission, cause of trauma, types of injuries, Glasgow Coma Score (GCS), and ISS were recorded on standardised forms. No patient had any comorbidity or chronic disease. The control group included 30 patients who had not suffered trauma.

### 2.2. Blood Samples

Blood samples were collected from all patients at admission to determine the MPV and the levels of CRP, IL1*β*, IL6, and TNF*α*. All assays were completed within 15 min in the emergency laboratory.

### 2.3. Measurements of Systemic Inflammatory Markers

CRP levels were first immunoturbidimetrically analysed on an Architect-Plus Ci 4100 instrument (Abbott Park, Chicago, IL, USA). Residual serum was stored at −80°C. Blood cell counts were obtained using a Cell-Dyn 3700 platform (Abbott Diagnostics, Santa Clara, CA, USA). Serum TNF*α*, IL1*β*, and IL6 levels were measured using Boster ELISA kits (Freemont, CA, USA) and Bio-Tek (Winooski, VT, USA) ELx50 and ELx800 devices.

### 2.4. Statistical Analysis

Data were analysed using Statistical Package for Social Sciences (SPSS) software, version 20.0. Parametrical values were compared using *t*-test, and the chi-squared test was employed to compare nonparametrical values. A *p* value < 0.05 was considered to reflect statistical significance.

## 3. Results

### 3.1. Clinical Characteristics of the Study Group

Of the 114 included patients, 33 had ISSs > 15, and 51 had ISSs ≤ 15. Thirty healthy volunteers formed the control group. The mean age of the 33 severely injured patients was 42.18 ± 15.04 years. The mean age of the 51 mildly injured patients was 38.75 ± 15.91 years. These ages did not significantly differ (*p* = 0.321).

The mean heart rate of severely injured patients was 87.73 ± 13.269 beats/min; that of mildly injured patients was 82.69 ± 11.513 beats/min; these values did not differ significantly (*p* = 0.078). The average systolic blood pressure of patients with ISSs > 15 was 139.30 ± 20.866 mmHg, which was significantly higher than that of patients with ISSs ≤ 15 (127.45 ± 18.010 mmHg; *p* = 0.009). However, the mean diastolic pressures did not differ significantly (*p* = 0.043), as they were 79.15 ± 13.311 and 72.65 ± 15.406 mmHg in the severely and mildly injured groups, respectively (Table 1).

Of the 84 study patients, 25 (29.7%) were hit by motor vehicles, 21 (25%) were inside motor vehicles that were in traffic accidents, 18 (21.4%) had motorcycle accidents, 12 (14.2%) had simple falls, and 8 (9.5%) fell from heights. The causes of trauma are listed in Table 2 and the injuries suffered are listed in Table 3.

### 3.2. Plasma Concentrations of Inflammatory Markers

The mean CRP value in the study group was 12.66 ± 7.71 mg/L, and it was 3.04 ± 0.98 mg/L in the control group. Injured patients thus had significantly higher CRP values (*p* = 0.003).

The mean MPV of the study group was 7.76 ± 1.11 fL, and that of the control group was 7.55 ± 1.02 fL. These values did not differ significantly (*p* = 0.347).

The mean TNF*α* value of the study group was 50.45 ± 22.15 pg/mL, and that of the control group was 25.04 ± 10.01 pg/mL. The TNF*α* level was thus significantly higher in the study group (*p* = 0.000).

The mean IL6 level was 70.66 ± 40.05 pg/mL in the study group, and that of the control group was 6.857 ± 5.07 pg/mL. The IL6 level was thus significantly higher in the study group (*p* = 0.000).

The mean IL1*β* value in the study group was 12.66 ± 7.71 pg/mL, and that of the control group was 3.04 ± 0.98 pg/mL. The IL1*β* level was thus significantly higher in the study group (*p* = 0.000).

The mean MPV value in the 33 patients with ISSs > 15 was 7.62 ± 1.19 fL; this value was 7.86 ± 1.06 fL in the 51 patients with ISSs ≤ 15. These values did not differ significantly (*p* = 0.355).

Patients with ISSs > 15 had a mean CRP level of 0.66 ± 0.84 mg/L; that of patients with ISSs ≤ 15 was 0.57 ± 1.26 mg/L. These values did not differ significantly (*p* = 0.693).

The mean TNF*α* level of patients with ISSs > 15 was 59.98 ± 18.40 pg/mL; that of patients with ISSs ≤ 15 was 44.43 ± 22.38 pg/mL. More severely injured patients had significantly higher TNF*α* values (*p* = 0.001).

Patients with ISSs > 15 had a mean IL1*β* value of 13.10 ± 8.10 pg/mL; that of mildly injured patients (ISSs ≤ 15) was 12.38 ± 7.52 pg/mL. These values did not differ significantly (*p* = 0.682).

The mean IL6 level in severely injured patients (ISSs > 15) was 88.43 ± 51.75 pg/mL; that of patients with milder injuries (ISSs ≤ 15) was 59.16 ± 24.63 pg/mL. The IL6 level was significantly higher in patients with severe injuries (*p* = 0.004). The comparisons of biochemical markers between the study and control groups are shown in Table 4.

## 4. Discussion

Although death rates have declined since the 1960s, unintentional injuries remain a major cause of death [1]. The World Health Association noted that traffic accidents are the most common causes of trauma [2]. We found this to be true, as 64 (76.1%) of our 84 study group patients were injured in traffic accidents; this proportion was higher than the generally reported because our hospital is a tertiary referral centre located near a major highway. Additionally, we excluded alcohol and drug abusers and patients with chronic diseases, which may have decreased the number of injuries caused by falls.

After trauma, hypothalamic activation of the sympathetic autonomous nerve system triggers norepinephrine secretion from presynaptic nerve terminals and catecholamine discharge from the adrenal medulla [4]. This raises the blood pressure. We found a significant difference in systolic blood pressure between patients with major and minor trauma. This was not the case for diastolic pressure, however. Systolic pressure rose as a response to injury, but diastolic pressure did not.

Although the MPV was previously reported to predict trauma severity [4], we found no significant difference between those who were mildly or severely injured or between those who were injured and controls.

A meta-analysis of the CRP levels of 13,374 patients revealed that an increase in that level was associated with trauma [5]. We also found a significant increase in CRP levels in injured patients; however, the ISSs did not correlate with these levels. A previous study found that CRP levels rose 6–12 h after trauma; thus, this parameter may not be useful in the initial screening of trauma severity [6].

TNF*α* is a proinflammatory cytokine synthesised by activated macrophages and T lymphocytes; TNF*α* induces vasodilatation and increases vascular permeability [7]. TNF*α* release is faster than that of cytokines, and the elevated TNF*α* levels after trauma are harmful [8]. TNF*α* triggers central sensitisation and hyperalgesia [8]. The TNF*α* level in the study group was significantly higher than that in the control group. Among severely and mildly injured patients, TNF*α* levels were higher in those with ISSs > 15, and the TNF*α* level correlated with trauma severity.

Trauma severity is correlated with ISSs, MODS scores, ARDS scores, and the incidence of sepsis [9, 10]. The IL6 level soon after trauma is associated with mortality and treatment response in animals [11]. In another study, higher IL6 levels were evident in patients with ISSs > 15 and were associated with increased mortality [12]. We found that IL6 levels were significantly higher in the study group, and patients with ISSs > 15 had higher IL6 levels than patients with ISSs ≤ 15. Trauma severity is thus associated with a higher IL6 level.

Release of IL1*β* concomitantly with TNF reduces pain sensations but causes fever, anorexia, polymorphonuclear leukocyte excretion, T lymphocyte proliferation, T lymphocyte transmigration to the area of injury, expression of adhesion molecules in the endothelium, and an increase in cell permeability [13]. One study found that IL6 and TNF*α* levels were associated with mortality, and IL8 level was associated with the ISS. However, the IL1*β* level was not related to the ISS in our current work, as the ISS was only mildly elevated at high levels of IL1*β*. Thus, in our study, IL1*β* level did not aid in the determination of trauma severity. Moreover, neither the MPV nor the CRP level was useful in this context.

## 5. Conclusion

Trauma mortality can be reduced by effective resuscitation, fast transport to a hospital, and effective trauma management in the emergency department. Such management begins with an accurate assessment of the patient. In the present study, we sought correlations between the levels of biochemical markers and ISS scores. The CRP level was elevated in all trauma patients, but that level did not correlate with the ISS. The IL1*β* level was elevated in all trauma patients, but it was not higher in patients with higher ISSs compared to those with lower ISSs. The IL6 and TNF*α* levels were higher in the study group and were correlated with ISSs. The MPVs did not differ between the trauma and control groups.

IL6 and TNF*α* levels can be used to assess trauma severity. However, the MPV and CRP and IL1*β* levels are not useful in this regard.

## Figures and Tables

**Table 1:** Vital signs.

	ISS > 15 (*n* = 33)	ISS ≤ 15 (*n* = 51)	*p*
Age	42 ± 15.04	38.75 ± 15.91	0.321
Heart rate	87.73 ± 13.269	82.69 ± 11.513	0.078
Systolic pressure	139.30 ± 20.866	127.45 ± 18.010	0.009
Diastolic pressure	75.15 ± 13.311	72.65 ± 15.40	0.043

**Table 2:** Reasons of trauma.

Motor vehicle hit	25 (29.7%)
Traffic accident while inside the vehicle	21 (25%)
Motorcycle accident	18 (21.4%)
Simple falls	12 (14.2%)
Fall from height	8 (9.5%)

**Table 3:** Injuries of the trauma patients.

Type of injury	Number of patients
Epidural bleeding	3
Subarachnoid bleeding	2
Vertebral fractures	3
Nasal fractures	4
Mandibular fractures	1
Maxillary fractures	1
Scapula fractures	1
Clavicle fractures	1
Costal fractures	9
Pneumothorax	4
Humerus fractures	1
Radius fractures	4
Pelvic fractures	3
Femur fractures	4
Tibial fractures	2
Calcaneal fractures	1

**Table 4:** Comparison of biochemical markers within between study groups and controls.

	Study group			Control group	*p*
MPV	7.76 ± 1.11			7.55 ± 1.02	0.347
	ISS > 15	ISS ≤ 15	*p*		
	7.62 ± 1.19	7.86 ± 1.06	0.355		

CRP	0.61 ± 1.11			0.22 ± 0.11	0.003
	ISS > 15	ISS ≤ 15	*p*		
	0.66 ± 0.84	0.57 ± 1.26	0.693		

TNF*α*	50.45 ± 22.15			25.04 ± 10.01	<0.005
	ISS > 15	ISS ≤ 15	*p*		
	59.98 ± 18.40	44.43 ± 22.38	0.001		

IL6	70.66 ± 40.05			6.857 ± 5.07	<0.005
	ISS > 15	ISS ≤ 15	*p*		
	88.43 ± 51.75	59.16 ± 24.63	0.004		

IL1	12.66 ± 7.71			3.04 ± 0.98	<0.005
	ISS > 15	ISS ≤ 15	*p*		
	13.10 ± 8.10	12.38 ± 7.52	0.682		
